# Spoof localized surface plasmons on ultrathin textured MIM ring resonator with enhanced resonances

**DOI:** 10.1038/srep14819

**Published:** 2015-09-30

**Authors:** Yong Jin Zhou, Qian Xun Xiao, Bao Jia Yang

**Affiliations:** 1 Key Laboratory of Specialty Fiber Optics and Optical Access Networks, Shanghai University, Shanghai 200072, China

## Abstract

We numerically demonstrate that spoof localized surface plasmons (LSPs) resonant modes can be enhanced based on ultrathin corrugated metal-insulator-metal (MIM) ring resonator. Further enhancement of the LSPs modes has been achieved by incorporating an efficient and ease-of-integration exciting method. Quality factors of resonance peaks have become much larger and multipolar resonances modes can be easily observed on the textured MIM ring resonator excited by a microstrip line. Experimental results validate the high-efficiency excitation and resonance enhancements of spoof LSPs modes on the MIM ring resonator in the microwave frequencies. We have shown that the fabricated resonator is sensitive to the variation of both the dielectric constant and the thickness of surrounding materials under test. The spoof plasmonic resonator can be used as key elements to provide many important device functionalities such as optical communications, signal processing, and spectral engineering in the plasmonic integration platform.

Surface plasmons (SPs) are surface electromagnetic (EM) waves confined at the interface of metal and dielectric at optical frequencies^1^, which can overcome diffraction limit due to strong subwavelength field confinement^2^. Surface plasmons exist either as propagating surface plasmon polaritons (SPPs) at the extended interface of metal and dielectric or as localized surface plasmons (LSPs) on small metal particles^3^. LSPs have been of interest for increasing applications in the optical antenna^4^,^5^, surface-enhanced Raman scattering^6^,^7^, chemical and biological sensors^8^,^9^,^10^, and photovoltaics^11^,^12^, etc. At terahertz (THz) and microwave frequencies, however, metals behave akin to perfect electric conductors which do not support SPPs.

In order to realize highly confined propagating surface waves at the microwave and THz frequencies, spoof (or designer) SPPs based on the structured metal surfaces were proposed^13^,^14^,^15^, paving the way to design and realize SPPs waveguides and components operating at lower spectral ranges^16^,^17^,^18^,^19^,^20^,^21^. Ultrathin corrugated metallic strips have been shown to support conformal SPPs, which open the way to the design of ultrathin microwave and THz plasmonic functional devices^22^,^23^,^24^,^25^,^26^,^27^. A pioneering study on spoof LSPs showed that a two dimensional (2D) periodically textured metallic cylinder could support spoof LSPs, and two interacting three dimensional (3D) textured cylinders could achieve field enhancement^28^. Spoof LSPs can also exist in a complementary structure–a periodically textured closed cavity^29^. Magnetic LSPs supported by cylindrical structures were then theoretically and experimentally explored, adding the important ingredient of magnetism to the field of particle plasmonics^30^. Recently, spoof LSPs on a planar textured metallic disk with nearly zero thickness were demonstrated experimentally at microwave frequencies^31^. Compared with long cylinders^28^, it is easy to observe dipole and quadrupole modes under the same background. High-order spoof LSPs modes in periodically corrugated metal particles were also investigated, showing these modes resemble the optical whispering gallery modes sustained by dielectric resonators^32^. However, not all the resonant modes can be clearly observable^31^. In the case of plane-wave excitation, the higher resonant modes are difficult to excite by a plane wave, while the dipole resonance is very weak under the excitation of the monopole source due to the mode mismatch. Although enhanced resonances of spoof LSPs on the ultrathin textured disk under the excitation of the monopole source have been experimentally demonstrated^33^, an extra ground plane underneath is necessary. Here we theoretically investigated and experimentally verify enhanced spoof LSPs resonances on ultrathin corrugated metal-insulator-metal (MIM) ring resonator without extra ground plane underneath.

MIM based plasmonic slot waveguides have been shown to provide both long-range propagation and subwavelength spatial confinement of light^34^,^35^,^36^. Among many MIM-based plasmonic components, ring resonator^37^,^38^,^39^,^40^ is a key element to implement plasmonic add-drop couplers^41^, filters^37^,^42^, wavelength-division-multiplexer^43^, refractive index sensor^44^, etc. 2D or ultrathin MIM waveguides which support spoof SPPs have been studied^23^,^45^,^46^,^47^,^48^, which are also called double gratings^23^, spoof-insulator-spoof (SIS) waveguide^46^, or complementary plasmonic metamaterial^48^. To the best of our knowledge, spoof LSPs on ultrathin MIM waveguides have not been reported. In this paper, the proposed ultrathin corrugated MIM ring resonator with enhance resonances is composed of two closed corrugated metal strips with periodic array of grooves. An efficient and ease-of-integration mechanism to excite spoof LSPs has been adopted to overcome the aforementioned excitation problems. It has been demonstrated that all the resonant modes have been further enhanced and quality (Q) factors of resonance peaks become much larger, indicating more sensitivity as a sensor. Both numerical simulations and experiments are conducted at microwave frequencies to verify the performance of the ultrathin corrugated MIM ring resonator excited by the microstrip line. We have demonstrated that the sensitivity of the fabricated sensor to the variation of dielectric constant is as high as 1.51 mm/RIU (Refraction Index Unit). Its sensitivity to the variation of the thickness of materials as thin as 1/67 wavelength can reach up to 1401. The spoof plasmonic resonator can be used as building block to implement many important applications such as optical communications, signal processing, and spectral engineering in the plasmonic integration platform.

## Results

### Design and dispersion relation of straight spoof plasmonic waveguides

**Figure 1:**
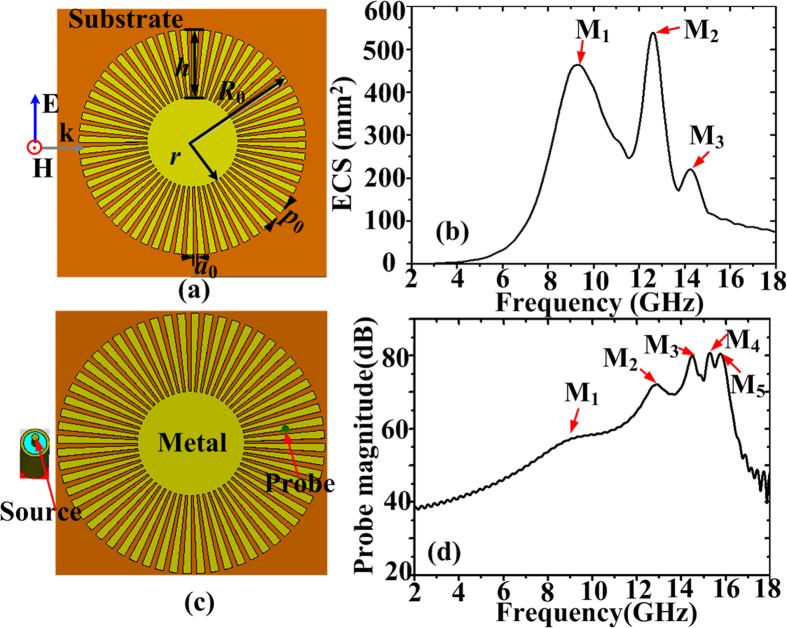
(**a**) Schematic diagram of the ultrathin textured metallic disk under the excitation of a plane wave. (**b**) The calculated ECS spectrum. The marked M_1_-M_3_ peaks correspond to dipole, quadrupole, and hexapole resonant modes, respectively. (**c**) Schematic diagram of the textured disk under the excitation of a monopole source. (**d**) The simulated near-field response, in which the marked red arrows from left to right indicate the M_1_–M_5_ resonance peaks, respectively.

First, we investigate the spoof LSPs supported by the ultrathin planar textured metallic disk depicted in Fig. 1(a)^31^, which is excited under a plane wave which is incident from the left to the right with a magnetic field perpendicular to the structure surface. The disk consists of an inner core of radius *r* surrounded by periodic array of grooves of pitch *p*_0_ = 2π*R*_0_/*N* (where *N* is the number of grooves). The groove height and groove width are *h* = *R*_0_ − *r* and *a*_0_ = 0.4*p*_0_. The disk is printed on the thin dielectric substrate (Rogers RO4350) whose thickness *t* and relative dielectric constant are 0.508 mm and 3.48, respectively. The parameters of the disk are set to be *R*_0_ = 5 mm, *r* = 2 mm, *N* = 60 (*p*_0_ = 0.52 mm), and *a*_0_ = 0.21 mm. The thickness of the metal is 0.018 mm. The calculated extinction cross section (ECS) spectrum is shown in Fig. 1(b), where three extinction peaks marked by M_1_-M_3_ can be observed. The disk is then excited by a monopole source and the probe is located at the opposite edge to the source and 0.5 mm above the textured disk, as illustrated in Fig. 1(c). The probed near-field response is displayed in Fig. 1(d), in which more resonance modes can be observed. In accordance with the previous study^31^, we can see that the higher resonant modes are difficult to excite by a plane wave, while the dipole resonance is very weak under the excitation of a monopole source.

**Figure 2:**
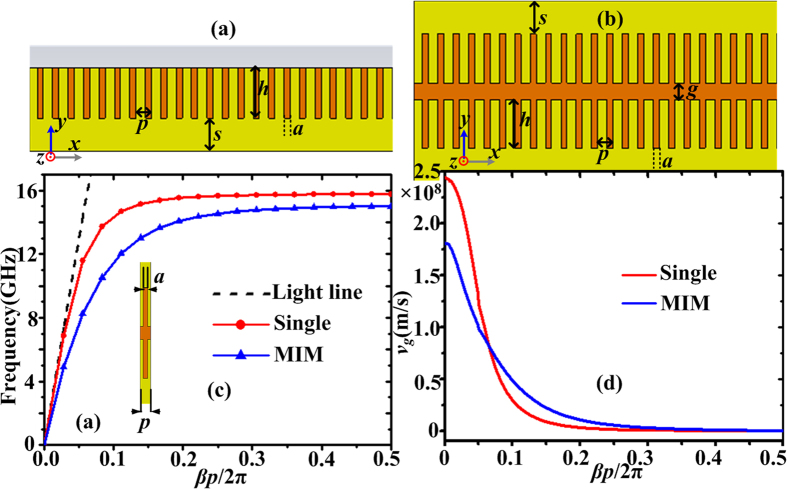
Schematic picture of (a) the single straight corrugated metallic strip and (b) the straight corrugated MIM waveguide. (**c**) The dispersion relations of spoof SPPs on the single or MIM corrugated strips. (**d**) The energy flow (group) velocity *v*_*g*_ of the spoof SPPs on the single or MIM corrugated strips.

To reveal the nature of spoof LSPs in the proposed spoof plasmonic resonator, we firstly investigate the EM characteristics of spoof SPPs on the straight spoof plasmonic structures, since the asymptotic frequency of a straight plasmonic structure can be maintained in the spoof plasmonic resonator. The textured disk arises from the single straight corrugated metallic strip shown in Fig. 2(a), while the proposed corrugated MIM ring resonator derives from straight corrugated MIM waveguide illustrated in Fig. 2(b). For simplicity, the insulator between the two metal strips is set as air. Here the parameters are *a* = 0.4*p*, *p* = 0.94 mm, *h* = 3 mm, *g* = 1 mm, and *s* = 2 mm. The dispersion relations of spoof SPPs on the single or MIM corrugated strips are calculated by use of the eigenmode solver of the commercial software, CST Microwave Studio. The dispersion curves are plotted in Fig. 2(c). Only one unit cell (see the inset) is used in the simulation. The energy flow (group) velocity *v*_*g*_ of the spoof SPPs can be calculated from the dispersion curves according to *v*_*g*_ = *dω*/*dβ* and the results are shown in Fig. 2(d). From these curves, we can obtain several conclusions. First, it can be seen that the asymptotic frequency of the corrugated MIM waveguide is lower than that of the single corrugated metallic strip. For the same operating frequency, the plasmonic wave vector *β* of spoof SPPs in the corrugated MIM strips becomes larger. Hence, stronger field confinement can be achieved in the corrugated MIM waveguide, since the SPP fields in the air fall off as 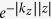 with 
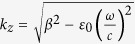
, where *k*_z_ is the space wave vector in *z*-direction (perpendicular to the metal surface), *ω* is the angular frequency, *ε*_0_ and *c* are the permittivity and light velocity in the air. Second, close to the asymptotic frequency, the dispersion curve for the corrugated MIM strips slowly becomes flat in a large wave-number range, rather than abruptly turning flat as in the case of single corrugated metallic strip. In other words, the group velocity *v*_*g*_ of the spoof SPPs in the corrugated MIM waveguide decreases more slowly than that in the single corrugated strip. The energy incident from the input port will be totally reflected before its group velocity decreases to zero, due to the strong intermodal coupling between the forward and backward modes^49^. Hence, if we define that *v*_*gmin*_ is the minimum group velocity where the energy flow velocity of the spoof SPPs has practically reached, there exists a cutoff wave-vector *β*_*max*_ where the dispersion curve has practically reached its asymptotic frequency^33^ and the *β*_*max*_ of the corrugated MIM strips will be larger than that of single corrugated metallic strip. Since only resonance modes with wave vectors smaller than *β*_*max*_ can form resonances, the number of resonance modes supported by the corrugated MIM ring resonator should be larger than that supported by the corrugated disk.

### Mode characteristics and excitation of spoof SPPs on straight plasmonic waveguides

**Figure 3:**
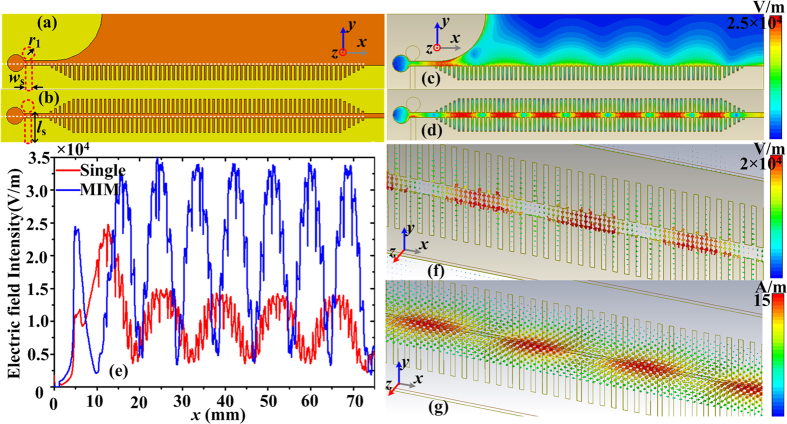
(**a**) Schematic diagram of the single straight corrugated strip excited by the microstrip line on the bottom of the substrate, where *w*_s_ = 1.1 mm, *l*_*s*_ = 6 mm, and *r*_1_ = 1.5 mm. (**b**) Schematic diagram of the straight corrugated MIM waveguide excited by the microstrip line. 2D electric-field distribution in the *xy*-plane at 8 GHz for the single corrugated strip (**c**) and for the corrugated MIM waveguide (**d**). (**e**) The electric fields varying on an observed line along the *x* direction, which is corresponding to the white dotted line in Fig. 3(**a,b**). (**f**) The electric fields in the *xy*-plane and (**g**) the magnetic fields in the *xz*-plane for the corrugated MIM waveguide at 8 GHz.

There is an important question of how to excite efficiently spoof SPPs on the spoof plasmonic waveguides. The launching device can be considered as a field transformer which converts the field of a waveguide into that of spoof SPPs. The efficiency will be greater if the field built up by the launching device has a better agreement to that of surface wave^50^. The ultrathin MIM waveguide without grooves is similar to the slot line which has been deeply investigated and widely used in microwave circuits. It’s been found that if a slot line and a microstrip line cross each other at right angles, coupling will be especially tight^51^ due to good magnetic field matching between the slot line and the microstrip line. Here a microstrip line is used to excite spoof SPPs on the straight single or MIM corrugated strips. The schematic configurations are displayed in Fig. 3(a,b), where the EM energies are fed by the microstrip line (see the red dashed line) on the bottom of the dielectric substrate. In order to minimize the reflected waves from the end of the microstrip line, the metallic disk with radius *r*_1_ = 1.5 mm is connected to the microstrip line to increase the coupling degree of EM energies^52^. The width *w*_*s*_ and length *l*_*s*_ of the microstrip line are 1.1 mm and 6 mm, respectively. The tapering part is also used to avoid reflection. First, we observe the 2D electric field distribution in the *xy*-plane at 8 GHz, as illustrated in Fig. 3(c,d). Figure 3(e) shows the electric fields varying on an observed line along the *x* direction, which is corresponding to the white dotted line in Fig. 3(a,b). It is clearly found that the electric fields in the corrugated MIM strips are much higher than those in single corrugated strip. This better field confinement is consistent with the dispersion analysis in Fig. 2(c). Besides, it has been shown that the loss of spoof SPPs in corrugated MIM waveguide is obviously lower than that in single corrugated metallic strip^23^. Q factor is 2*π* times the ratio of the total energy stored divided by the energy lost in a single cycle. The larger the total energy stored is or the smaller the energy loss is, the larger Q factor is. Hence, it’s expected that Q factor of resonance modes in the corrugated MIM ring structure should be larger than that in the corrugated disk. Second, the electric fields on the *xy*-plane and the magnetic fields on the *xz*-plane of the corrugated MIM waveguide at 8 GHz are demonstrated in Fig. 3(f,g). Firstly, the dominant surface wave mode on the corrugated MIM waveguide is anti-symmetric (odd mode), which has been reported earlier^48^. Secondly, the major electric field component is oriented across the insulator (air) and the longitudinal component of the electric field is very weak. The major magnetic field component is vertical to the insulator surface, as shown in Fig. 3(g). After figuring out the EM field distributions on the corrugated MIM waveguide, we can understand why spoof LSPs in the corrugated MIM ring can be excited by different excitation sources in the following section. The exciting efficiency depends on the matching degree between the magnetic field of the exciting source and that in the corrugated MIM ring.

### Spoof LSPs on the corrugated MIM ring structure

**Figure 4:**
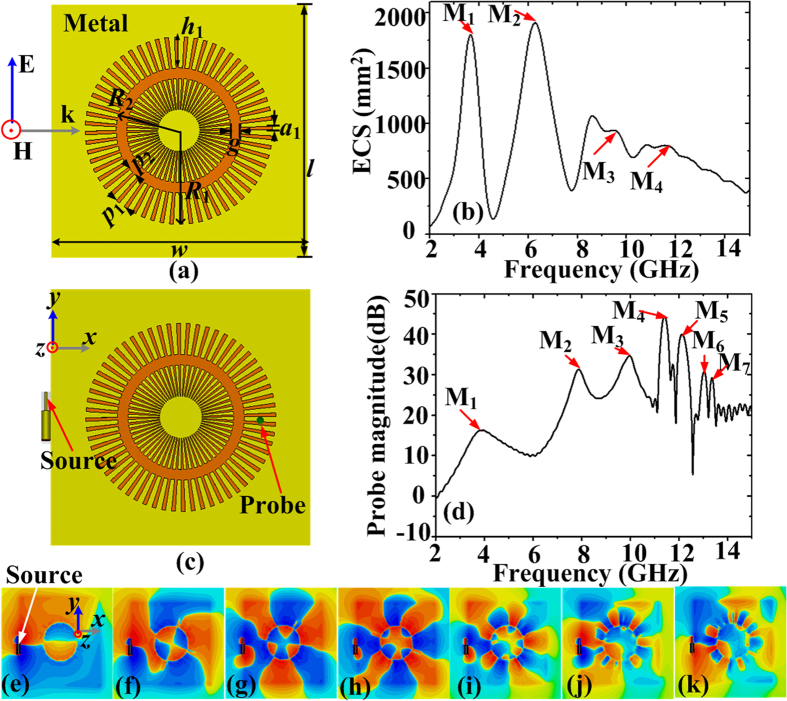
(**a**) Schematic picture of the proposed corrugated MIM ring resonator under the excitation of a plane wave. (**b**) The calculated ECS spectra. (**c**) Schematic picture of the corrugated MIM ring resonator under the excitation of a monopole source. (**d**) The probed near-field response spectra. (**e**–**k**) The 2D electric-field distributions on the plane 0.5 mm above the MIM ring resonator at the resonant frequencies marked by M_1_-M_7_, which are located at 3.87, 7.89, 9.97, 11.4, 12.13, 13.02, and 13.4 GHz.

Figure 4(a) depicts the schematic configuration of the proposed ultrathin corrugated MIM ring resonator, which is composed of two closed corrugated metal strips. The parameters of the inner corrugated metallic disk are the same as those of the disk in Fig. 1(a). The outer radius *R*_1_ and the inner radius *R*_2_ of the opposite closed corrugated metal are set to be 9 mm and 6 mm, respectively. The groove depth, groove period and groove width of the outer closed corrugated metal grating are denoted by *h*_1_, *p*_1_, *a*_1_, and they are set to be *h*_1_ = *h* = 3 mm, *p*_1_ = 2π*R*_1_/*N* = 0.94 mm, and *a*_1_ = 0.4*p*_1_. Both the width *w* and the length *l* of the whole structure are 25 mm. The width *g* of the air is optimized to 1 mm. The optimization mechanism will be discussed in the later section. Figure 4(b) provides the simulated ECS spectrum of the ultrathin corrugated MIM ring resonator under the excitation of a plane wave, where marked M_1_-M_4_ peaks correspond to multiple plasmonic resonances with the dipole, quadrupole, hexapole, and octopole modes at frequencies 3.64, 6.31, 9.55, and 11.71 GHz, respectively. To excite the spoof LSPs by use of a monopole source, the monopole should be parallel to the structure surface, as depicted in Fig. 4(c), to make sure that the magnetic field generated by the monopole source is perpendicular to the structure surface. Hence the magnetic field matches that of spoof LSPs in the corrugated ring structure. The near-field response is plotted in Fig. 4(d), in which seven peaks M_1_-M_7_ located at 3.87, 7.89, 9.97, 11.4, 12.13, 13.02, and 13.4 GHz can be observed. Several conclusions can be drawn by comparing Fig. 4 with Fig. 1. First, more resonance modes in the corrugated MIM ring resonator can be observed, no matter a plane wave or a monopole source is used to excite spoof LSPs. The result verifies the theoretical analysis in Fig. 2(c,d). Second, the resonances have been enhanced, which is consistent with the analysis in Fig. 3(c–e). For example, for the case under the excitation of a plane wave, Q factor of M_1_ resonance peak has been increased a little, changing from 2.7 to 4.2. For the case under the excitation of a monopole source, the dipole resonance is obviously enhanced, although its Q factor is still only 3.17. Q factor of M_4_ resonance peak has been increased from 40.4 to 54.28. Third, spoof LSPs in the corrugated MIM ring structure are actually standing surface waves^31^. To form resonances in the circular resonator, the well-known requirement to be satisfied is *L* = *nλ*_*g*_, where *L* is the circumference of the circular resonator, *λ*_*g*_ is the guided wavelength on the straight corrugated MIM waveguide, and *n* is a positive integer. Since groove width of the corrugated MIM ring resonator is non-uniform, we choose a medium groove width to check whether the above relation is satisfied. Hence *L* = 2π*R*_2_ = 37.7 mm and the groove width is *a*_2_ = 0.4*p*_2_, where *p*_2_ = 2π*R*_2_/*N* = 0.63 mm. The wavelength at the resonant modes marked by M_1_-M_7_ can be calculated by *λ*_*g*_ = 2π/*β*. The calculated results are *λ*_*g*M1_ = 39.1 mm, *λ*_*g*M2_ = 17.2 mm, *λ*_*g*M3_ = 12.2 mm, *λ*_*g*M4_ = 9.3 mm, *λ*_*g*M5_ = 8.0 mm, *λ*_*g*M6_ = 6.6 mm, and *λ*_*g*M7_ = 6.0 mm. It can be verified that the circumference *L* approximately satisfy *L* ≈ M_*i*_ × *λ*_*g*M*i*_, (*i* = 1, 2, 3……). Lastly, comparing Fig. 4(d) with Fig. 4(b), we can see that the higher modes are also difficult to excite by a plane wave due to the low matching between the field of the plane wave and that of the spoof LSPs for higher resonant modes. In order to verify the multipolar spoof LSPs resonant modes on the corrugated MIM structure excited by the monopole antenna, 2D distributions of electric-field (*z*-components) on the plane 0.5 mm above the corrugated MIM ring resonator at the resonant frequencies are illustrated in Fig. 4(e–k), where the color scale ranges from red (the highest positive intensity) to blue (the lowest negative intensity). It can be seen that all the resonance modes at M_1_-M_7_ can be recognized, while the field patterns in M_6_ and M_7_ are not clearly observable.

**Figure 5:**
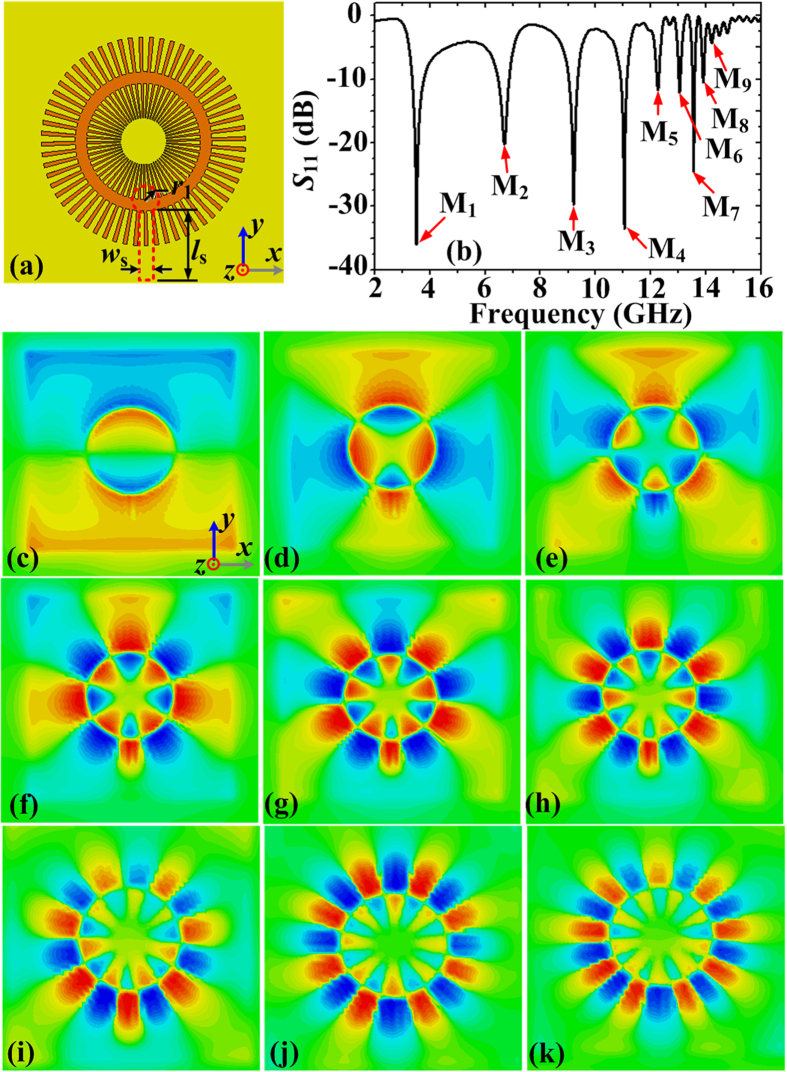
(**a**) Schematic picture of the ultrathin MIM ring resonator excited by the microstrip line at the bottom of the dielectric substrate. (**b**) The simulated reflection coefficients (*S*_11_) of the corrugated MIM ring resonator excited by the microstrip line. The M_1_-M_9_ nadirs correspond to nine resonant modes, which are located at 3.52, 6.7, 9.2, 11.04, 12.26, 13.06, 13.57, 13.9, and 14.21 GHz, respectively. (**c**–**k**) The near electric-field patterns in the plane 0.5 mm above the corrugated MIM resonator at the M_1_-M_9_ nadirs.

Next a microstrip line is used to excite the LSPs modes to further improve the performance of the ultrathin corrugated MIM ring resonator and the schematic configuration is displayed in Fig. 5(a). The parameters of the microstrip are the same as those in Fig. 3(a). The reflection coefficients *S*_11_ presented in Fig. 5(b) demonstrate that the dipole mode (M_1_) and all higher modes (M_2_–M_9_) are obviously enhanced with higher Q factors. For examples, Q factor of M_1_ resonance peak has been increased to 70.4, while it’s only 3.17 for the case under the excitation of a monopole source. Q factor of M_4_ resonance peak has been changed from 54.28 to 184. Especially, the higher modes M_8_ and M_9_ that were absent in Fig. 4(d) can also be observed. The resonance peaks of these modes are located at 3.52, 6.7, 9.2, 11.04, 12.26, 13.06, 13.57, 13.9, and 14.21 GHz, respectively. Compared to the resonance peaks M_1_–M_4_ located at 3.64, 6.31, 9.55, and 11.71 GHz for the case under the excitation of a plane wave, there are little deviations. To visualize the resonant modes clearly, the simulated near-field distributions on the plane 0.5 mm above the corrugated MIM ring resonator are illustrated in Fig. 5(c–k), which are corresponding to the M_1_–M_9_ resonance modes, respectively. Comparing with Fig. 4(e–k), we can find that all the field patterns in M_1_-M_9_ can be more clearly observable.

**Figure 6:**
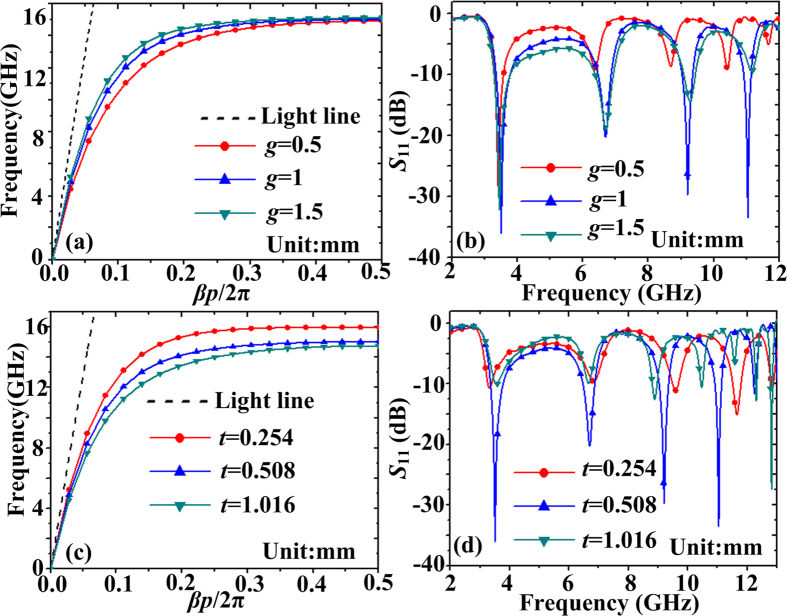
(**a**) Dispersion curves of spoof SPPs on the corrugated MIM strips when the width *g* of the insulator (air) changes, where *p* = 0.94 mm, *a* = 0.376 mm, *h* = 3 mm, and *s* = 2 mm for one unit cell. (**b**) Reflection coefficients (*S*_11_) spectra of corrugated MIM resonator with different insulator widths. (**c**) Dispersion curves of spoof SPPs for the corrugated MIM strips when the thickness *t* of the substrate changes. (**d**) *S*_11_ spectra of corrugated MIM resonator with different thickness *t* of the substrate.

When the thickness *t* of the substrate and the width *g* of the insulator (air) changes, the dispersion relations of corresponding spoof SPPs on the corrugated MIM strips are plotted in Fig. 6(a), where *p* = 0.94 mm, *a* = 0.376 mm, *h* = 3 mm, and *s* = 2 mm for the unit cell in simulation. When the width *g* of insulator (air) is changed from 1.5 mm to 0.5 mm, corresponding asymptotic frequencies of dispersion curves in Fig. 6(a) are pulled down. Reflection coefficients (*S*_11_) spectra of corrugated MIM resonator with different insulator widths are shown in Fig. 6(b). It can be seen that all resonance nadirs below asymptotic frequencies red shift when *g* decreases. According to the above standing wave analysis in Fig. 4(d), for a specific resonant mode M_*i*_, the spoof LSPs wavelength *λ*_*g*M*i*_ and wave vector *β* are fixed. From the dispersion curves in Fig. 6(a), for the same plasmonic wavevector *β*, the frequency becomes smaller. Hence, the red shifts of spoof LSPs resonant frequencies are consistent with the dispersion relations. When the thickness *t* of substrate is gradually increased from 0.254 mm to 1.016 mm, corresponding asymptotic frequencies of dispersion curves shown in Fig. 6(c) are pulled down. Red shift of resonance nadirs below asymptotic frequencies can also be observed from *S*_11_ spectra with different thickness *t*, which are plotted in Fig. 6(d). From Fig. 6(b,d), we can see that nearly all the resonance modes are the strongest when *g* = 1 mm or *t* = 0.508 mm. Hence the optimized parameters are chosen as *g* = 1 mm and *t* = 0.508 mm.

### Experimental results

**Figure 7:**
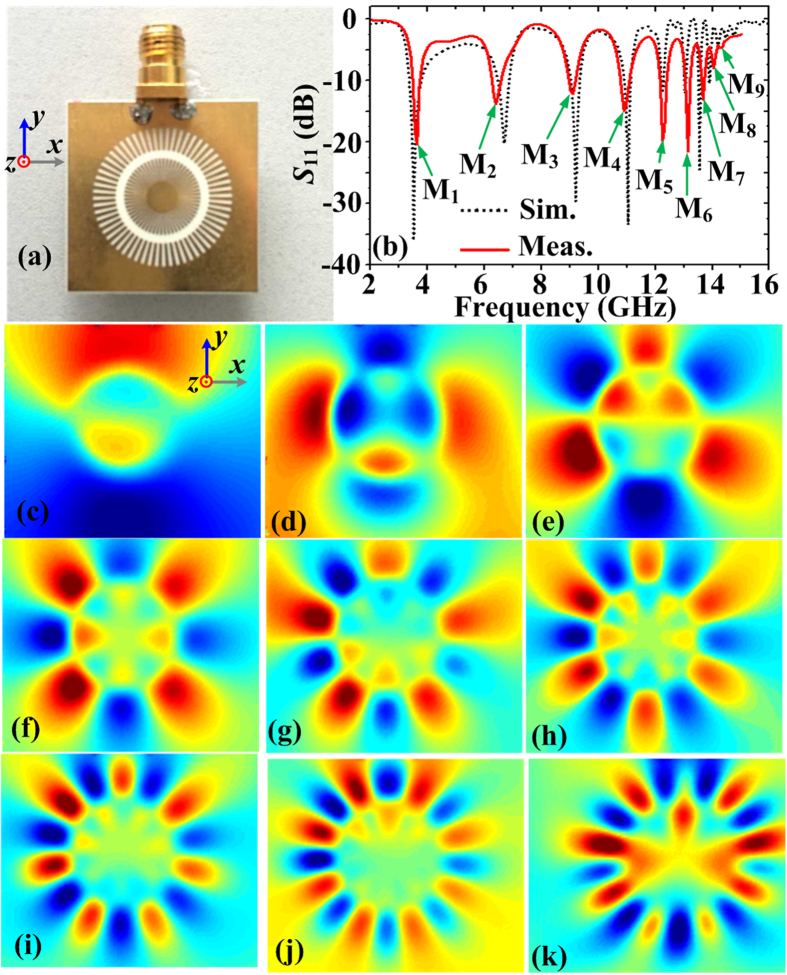
(**a**) The photograph of the fabricated MIM ring resonator on a dielectric substrate. (**b**) The simulation and measurement *S*_11_ curves. (**c**–**k**) The measurement results of near electric-field patterns in the plane 0.5 mm above the fabricated sample at the resonant modes M_1_-M_9_, which are located at 3.58, 6.34, 9.09, 10.95, 12.28, 13.11, 13.64, 14.05, and 14.28 GHz, respectively.

To verify experimentally spoof LSPs on the corrugated MIM ring resonator with enhanced resonances, we have fabricated the sample shown in Fig. 7(a). The measured and simulated reflection coefficients spectrums are demonstrated in Fig. 7(b), where the black dashed line and red solid line correspond to simulation and measurement *S*_11_ curves, respectively. The green arrows indicate the measured resonance peaks at M_1_-M_9_ modes, which are located at 3.58, 6.34, 9.09, 10.95, 12.28, 13.11, 13.64, 14.05, and 14.28 GHz, respectively. Compared to the simulated resonant frequencies located at 3.52, 6.7, 9.2, 11.04, 12.26, 13.06, 13.57, 13.9, and 14.21 GHz, there are little deviations in the range of allowable error. For further validations, the measured near-field distribution on the plane 0.5 mm above the fabricated sample at these resonant frequencies are illustrated in Fig. 7(c–k). Comparing Fig. 5 with Fig. 7, we have confirmed that both the resonant frequencies and near-field patterns have good agreements between the simulations and measurements.

### Impact of surroundings on spoof LSPs resonances

**Figure 8:**
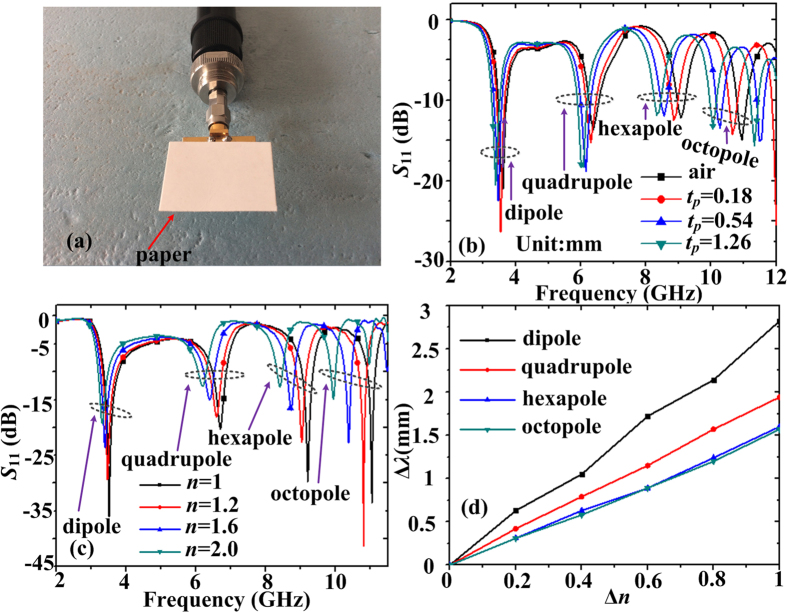
(**a**) The fabricated sample covered by thin paper card with different thickness. (**b**) The change of measured *S*_11_ curves with different thickness of thin paper cards. (**c**) Redshifts of spoof LSPs resonant frequencies when the whole MIM ring resonator is covered by detected materials with increasing indices of refraction. (**d**) The dependence of the variation of peak wavelength on the variation of the refractive index of the detected materials.

We have found that the spoof LSPs resonant modes are sensitive to the variation of the thickness of thin paper card. Here the thin paper card is put on the surface of the resonator, as shown in Fig. 8(a). The thickness *t*_*p*_ of the paper card under test is changed from 0.18 mm to 1.26 mm. The measured reflection coefficients (*S*_11_) are plotted in Fig. 8(b), where *t*_*p*_ = 0.18 mm, 0.54 mm, and 1.26 mm, respectively. We can observe that the resonant frequency shifts from 3.58 GHz to 3.41 GHz for the dipole mode, shifts from 6.34 GHz to 6.06 GHz for the quadrupole mode, shifts from 9.09 GHz to 8.34 GHz for the hexapole mode, and shifts from 10.95 GHz to 10.11 GHz for octopole mode. It means that we can obtain a 0.17 GHz (or 4.7%) shift in dipole resonance, a 0.28 GHz (or 4.4%) shift in quadrupole resonance, a 0.75 GHz (or 8.2%) shift in hexapole resonance, and 0.84 GHz (or 7.7%) shift in octopole resonance when the variation of the thickness of the paper card is 1.26 mm. The variations of the peak wavelengths correspond to 1765 mm, 1071 mm, 400 mm, and 357 mm, respectively. The sensitivity can be defined as *S* = Δ*λ*/Δ*t*_*p*_ and the sensitivities for dipole, quadrupole, hexapole, and octopole resonant modes are 1401, 850, 317, and 283, respectively. When the whole MIM ring resonator is covered by materials under test with different indices of refraction, shifts of all spoof LSPs resonant frequencies can also be observed. Figure 8(c) shows the simulated reflection coefficients when the refractive index of the detected material put on the resonator changes from 1.0 to 2.0. The dependence of the variation of peak wavelength on Δ*n* shows a nearly linear function, as shown in Fig. 8(d). The sensitivity is defined as *S* = Δ*λ*_*ave*_/Δ*n*, where Δ*λ*_*ave*_ is the average peak wavelength shift. Hence the sensitivities for dipole, quadrupole, hexapole, and octopole resonant modes are 2.83, 1.98, 1.55, and 1.51 mm/RIU, that is, nearly 1/30, 1/23, 1/21, and 1/18 wavelength RIU^−1^. Hence the ultrathin MIM ring resonator is sensitive to the surrounding refraction index and thickness variations.

## Discussion

In summary, we have numerically demonstrated that not only does the ultrathin corrugated MIM ring resonator allow for generation of higher number of spoof LSPs resonance modes, but it is also able to enhance resonant modes with higher Q factors, comparing with the corrugated metallic disk. Both numerical simulations and experimental results at microwave frequencies have shown that Q factors of all resonance modes have been further increased under the excitation of the microstrip line. The near field distributions at these resonant frequencies can be clearly observed, including higher-order modes M_8_ and M_9_ that were absent for the case under the excitation of a monopole source. It is also shown that the fabricated resonator is very sensitive to the variation of both dielectric constants and the thickness of surrounding materials. The spoof plasmonic resonator can find potential applications such as optical communications, signal processing, and spectral engineering in the plasmonic integration platform.

## Methods

### Simulation

The numerical simulations are conducted with the help of the commercial software, CST Microwave Studio. The recommended relative dielectric constant of Rogers RO4350 substrate during simulation is 3.66, with loss tangent 0.004. And its process specification value is 3.48. The calculation of dispersion relations of the textured disk or MIM ring resonator is based on CST eigenmode solver, where only one unit cell is analyzed and periodic boundary conditions (PBC) are used. ECSs, near electric field response, and the distributions of surface electric field of the textured disk or MIM ring resonator are calculated by use of CST transient solver, which is based on the finite-integral time-domain (FIT) method. Tetrahedral mesh and open boundary conditions are adopted. For ECSs calculation, a horizontal plane-wave excitation and a broadband far-field/RCS monitor to record the field information with 1000 frequency samples are applied. Under the excitation of the monopole source or the microstrip line, waveguide ports are used to generate the corresponding electromagnetic modes distributions.

### Experimental setup and measurement

**Figure 9:**
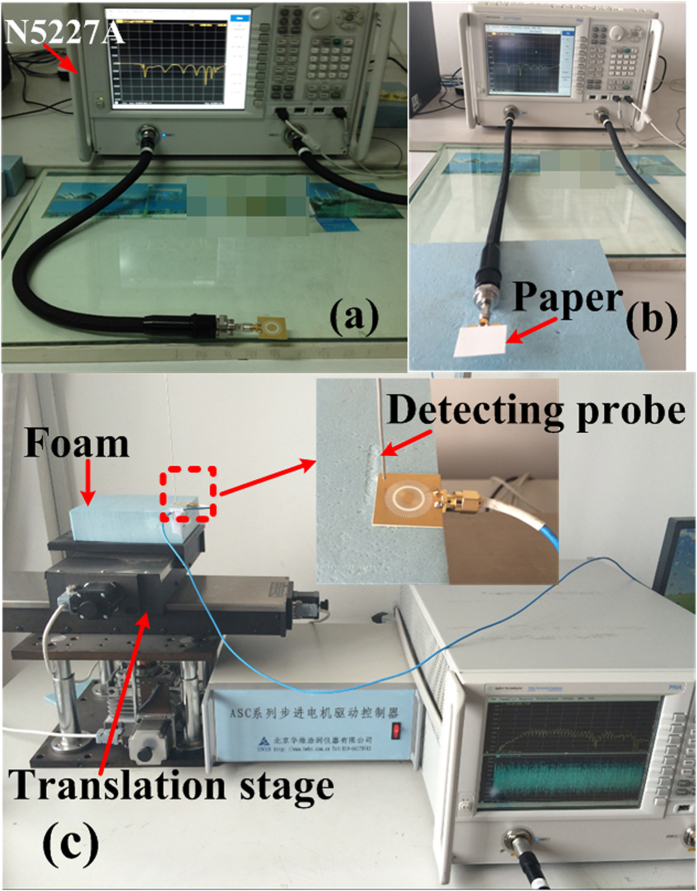
Experimental setup to measure the *S*_11_ parameters of (a) the fabricated sample and (b) the sample covered by thin paper card. (**c**) Photograph of the experimental platform to measure near electric-field distributions within the plane 0.5 mm above the sample. The setup consists in a vector network analyzer, an SFT-50-1 cable with a 0.2-mm-diameter inner conductor as the detector, coaxial cables, and a motion controller. The inset is an enlarged photograph of the sample under test in the dashed box the sample, showing the detailed arrangements of the detecting probe, feeding cable and the sample.

The fabricated sample is connected to Vector Network Analyzer (VNA, Agilent N5227A) to obtain the reflection coefficients, as illustrated in Fig. 9(a). The materials under test are put on the sample, as shown in Fig. 9(b). Figure 9(c) demonstrates the whole experimental setup to measure the distributions of the surface electric fields on the MIM ring resonator sample. The experimental setup consists of an Agilent N5227A, translation stages, coaxial lines, and a monopole antenna as detector. The sample is pasted on the foam which is mounted to two computer-controlled linear translation stages, enabling a scanning area of 26 mm by 24 mm with a resolution of 0.2 mm. The inner conductor of the detecting probe (SFT-50-1 cable) is extended 1 mm in order to sample the *z*-component of the electric fields within the plane 0.5 mm above the sample. The coaxial detecting probe is fixed onto the stationary shelf.

## Additional Information

**How to cite this article**: Zhou, Y. J. *et al.* Spoof localized surface plasmons on ultrathin textured MIM ring resonator with enhanced resonances. *Sci. Rep.* **5**, 14819; doi: 10.1038/srep14819 (2015).
